# TUBERCULOUS SARCOIDOSIS: DOES IT EXIST?

**DOI:** 10.4103/0970-2113.44140

**Published:** 2008

**Authors:** Ramakant Dixit

**Affiliations:** Department of Respiratory Medicine and Tuberculosis, J.L.N. Medical College, Ajmer., India

Dear Sir,

I read with interest the article ‘Tuberculous Sarcoidosis’ by Shah JR^1^ published in the recent issue (July-September 2007) of the Lung India. Author has discussed a possible association of two distinct clinical entities i.e. tuberculosis and sarcoidosis that may pose diagnostic & therapeutic problem and also described two such case histories of his experience. While there is no doubt in the second case, the first case requires further diagnostic workup for sarcoidosis i.e. SACE, serum calcium, urinary calcium, tissue biopsy & BAL analysis etc. to support clinical diagnosis of ‘tuberculous sarcoidosis’. Though in clinical practice, I fully agree that sometime complete diagnostic workup is not possible & conclusions are made after therapeutic response with drugs, which I do think is justified in our conditions at times.

Author has stressed on considering ‘tuberculous sarcoidosis’ as a definite clinical entity where two diseases present simultaneously or another disease develop in a course of existent disease. I thanks the author, for awaring the clinicians about this distinct entity for personal reason, that now I can suspect retrospective diagnosis of ‘tuberculous sarcoidosis’ in three cases in my previous clinical experience. Two AFB smear positive and tuberculin reactive pulmonary cases with noncaseating granulomas on tissue biopsy, required oral corticosteroid in view of poor response with antituberculosis drugs and another case, a 10 year old male child with lymphocyte predominant exudative pleural effusion and tuberculin reactivity developed cervical lymphadenopathy, nine months after completion of six months antituberculosis therapy with adequate response. The scalene node biopsy showed noncaseating granulomas and a second course with antituberculosis drugs resulted in poor response and patient lost to follow up. Although none of these cases could be investigated for sarcoidosis as diagnosis of tuberculosis was confirmed and the term ‘tuberculous sarcoidosis’ was neither familiar to me nor is mentioned in the standard textbooks of respiratory medicine till date. Since tuberculosis is rampant in our country and may present with various unusual clinical and radiological features, the diagnosis of other coexistent diseases is often overlooked. Lack of infrastructure, financial constraints, and lack of newer diagnostic techniques at most centre of our country, despite adequate clinical material and trained medical persons are possible causes for these situations. The recent studies on possible association of tuberculosis and sarcoidosis are interesting and encouraging further research in this field.

Sarcoidosis has pathologic^2^, immunologic^3^ and epidemiologic^4^ features that are similar to bacterial infection, particularly tuberculosis. While no infectious agent has been identified within sarcoidosis lesions, there are immunological and clinical aspects that suggest an infectious origin. Sarcoidosis immunology reflects Th-l cytokine expression, which is based upon antigen specific T-cell response. Studies of T-cell receptor gene expression in sarcoidosis patients reveal oligoclonal collections of αβ^+^ CD4+ T cells at the sites of granulomatous inflammation, consistent with a MHC-restricted antigen driven process^5^. Another feature is the transmissibility of sarcoidosis. There are reports of ‘Donor-acquired sarcoidosis’ in presumably naive (non sarcoidosis) transplant recipients who have received tissues or organs from donors having suspected or active sarcoidosis^6^. Recently, epidemiological studies have also suggested a possible role of certain occupation i.e. an agricultural employment, insecticide exposures and moldy environments that contains opportunistic mycobacterias^7^.

One of the strongest arguments against a potential role of mycobacteria in sarcoidosis pathogenesis is inability to detect microorganism on histological staining or by culture from pathologic tissues. The possible explanations for negative microbial workup noted in sarcoid lesions could be very low concentration of the bacteria's, ultra-slow growth pattern, and lack of certain nutritional requirements etc. or the sarcoidosis pathogenesis may reflect an immune response to infectious antigens that might not be dependent upon actively replicating organisms. Recently newer diagnostic techniques have been emerged for assessing presence of microorganisms in pathologic tissues. Polymerase chain reaction (PCR) analysis of pathologic tissue for 16S r RNA serve as an alternative means of identifying putative infectious agents that are difficult to isolate. One study of PCR analysis for conserved Mycobacterium 16S r RNA and rpo B sequences revealed the presence of slow-growing mycobacteria such as M tuberculosis and M avium, as well as unique 16S r RNA and rpo B sequences that suggest a novel mycobacterium^8^. Another study used spolingotyping of bronchoalveolar fluid and demonstrated the presence of unique sequence among 50% of sarcoidosis subjects, as well as the presence of sequence consistent with M.tuberculosis^9^. However, the limitation of PCR studies is that cross contamination may cause false positive results.

Another mechanism to identify the causative microorganism is use of antigen specific immune responses to microbial antigens and this has been. recently utilized to identify novel infectious agent in SARS^10^. A landmark study by Song et al^11^ involved molecular and immunologic analysis of sarcoidosis specimens for M tuberculosis katG. They demonstrated the presence of M tuberculosis katG in sarcoidosis granulomas by mass spectrophotometry and in-situ hybridization, as well as the presence of humoral immune response to M tuberculosis katG among sarcoidosis subjects. Another recent study has demonstrated a humoral response to M tuberculosis heat shock protein 70 in significant number of sarcoidosis patients^12^.

These recent studies of successful molecular analysis and humoral immunity to mycobacterial antigens from sarcoidosis patients have renewed interest in a potential role of mycobacteria in sarcoidosis and support the hypothesis that mycobacterias may have a causal role in ‘some’ sarcoidosis patients. Further molecular studies with positive and negative controls and investigation of genetic risk factors will be important, in order to explain why some patients are found to have an association with microbial antigens and others are not. In conclusion, we must be flexible on the diagnosis of two co existent diseases and possibility of ‘tuberculous sarcoidosis’ may be considered whenever such clinical situation arises and supported by complete investigation backup.
